# Supplementary material for: Genotyping by sequencing for the construction of oil palm (Elaeis guineensis Jacq.) genetic linkage map and mapping of yield related quantitative trait loci
Source: PeerJ. 2024 Jan 30;12:e16570. doi: 10.7717/peerj.16570 (PMC10836210; doi:10.7717/peerj.16570)
Supplement: Supplemental Information 2 [file peerj-12-16570-s002.docx]

**Supplementary 2:**

**The segregation patterns of SNP and InDel markers of 112 progenis from Deli *dura* and Serdang *pisifera* cross.**

A total of 5,278 markers, comprising 4,838 SNPs (91.7%) and 440 InDels (8.3%) were used to construct the genetic linkage map for Deli *dura* x Serdang *pisifera*.

| Segregation pattern | SNPs | Percentage (%) | InDels | Percentage (%) |
| --- | --- | --- | --- | --- |
| abxcd | 0 | 0.0 | 0 | 0.0 |
| efxeg | 0 | 0.0 | 2 | 0.5 |
| hkxhk | 9 | 0.2 | 13 | 3.0 |
| lmxll | 1,605 | 33.2 | 125 | 28.4 |
| nnxnp | 3,224 | 66.6 | 300 | 68.2 |
| Total | 4,838 | 100.0 | 440 | 100.0 |
